# The Neuronal Correlates of Digits Backward Are Revealed by Voxel-Based Morphometry and Resting-State Functional Connectivity Analyses

**DOI:** 10.1371/journal.pone.0031877

**Published:** 2012-02-16

**Authors:** Rui Li, Wen Qin, Yunting Zhang, Tianzi Jiang, Chunshui Yu

**Affiliations:** 1 Department of Radiology, Tianjin Medical University General Hospital, Tianjin, China; 2 LIAMA Center for Computational Medicine, National Laboratory of Pattern Recognition, Institute of Automation, Chinese Academy of Sciences, Beijing, China; National Research & Technology Council, Argentina

## Abstract

Digits backward (DB) is a widely used neuropsychological measure that is believed to be a simple and effective index of the capacity of the verbal working memory. However, its neural correlates remain elusive. The aim of this study is to investigate the neural correlates of DB in 299 healthy young adults by combining voxel-based morphometry (VBM) and resting-state functional connectivity (rsFC) analyses. The VBM analysis showed positive correlations between the DB scores and the gray matter volumes in the right anterior superior temporal gyrus (STG), the right posterior STG, the left inferior frontal gyrus and the left Rolandic operculum, which are four critical areas in the auditory phonological loop of the verbal working memory. Voxel-based correlation analysis was then performed between the positive rsFCs of these four clusters and the DB scores. We found that the DB scores were positively correlated with the rsFCs within the salience network (SN), that is, between the right anterior STG, the dorsal anterior cingulate cortex and the right fronto-insular cortex. We also found that the DB scores were negatively correlated with the rsFC within an anti-correlation network of the SN, between the right posterior STG and the left posterior insula. Our findings suggest that DB performance is related to the structural and functional organizations of the brain areas that are involved in the auditory phonological loop and the SN.

## Introduction

Working memory (WM) refers to a limited system that provides for the temporary storage and manipulation of the information necessary for complex cognitive tasks and that provides an interface between perception, long-term memory and action [1], [2]. The definition of WM has evolved from the concept of short-term memory but is defined in three different ways: as short-term memory applied to cognitive tasks; as a multi-component system that holds and manipulates the information in the short-term memory; and as the use of attention to manage the short-term memory [3]. A widely accepted model of WM has proposed that it consists of four subsystems, including the central executive system, the phonological loop, the visuospatial sketchpad, and the episodic buffer [1], [2], [4]–[6]. The phonological loop is specialized for processing verbal materials and is assumed to be a crucial component of the WM system for language acquisition [7]. Moreover, the phonological loop includes two subsystems: a phonological store, which has a limited information capacity and temporal trace (information can be held for a few seconds before it fades); and a subvocal rehearsal system, which continually repeats information to revive the memory trace in WM [2], [6]. The visuospatial sketchpad is a parallel to the phonological loop but exists and serves for the processing of visual and spatial information. The central executive system is an attentional control system that is responsible for strategy selection and for the regulation and coordination of the various processes involved in the phonological loop and the visuospatial sketchpad [2], [6]. The episodic buffer is assumed to be a limited-capacity system that depends heavily on executive processing but that differs from the central executive system in being principally concerned with the storage of information rather than with attentional control. The episodic buffer is capable of binding information from different modalities into a single multi-faceted code [7].

Most of our knowledge of the neural correlates of WM is derived from lesion studies and functional imaging studies, which have revealed that the phonological store depends largely upon the left inferior parietal cortex [8], [9]; the rehearsal processes are based on the left inferior frontal gyrus (IFG) (typically described as Broca's area), the premotor area and the supplementary motor area [10]–[14]; and the central executive system relies heavily on the frontal lobe, particularly the dorsolateral prefrontal cortex (DLPFC) and the dorsal anterior cingulate cortex (ACC) [15]. Although functional imaging can be used to identify the brain regions engaged in WM, it cannot be used to identify the neural correlates of WM capacity because functional imaging measures active processing, whereas capacity is a constraint on processing and not a process itself [16].

WM capacity is typically assessed using behavioral measures such as the digit-span test, in which participants are asked to perform the immediate recall of digit sequences of increasing length. Digits forward (DF) has been characterized as a simple span test and is thought to measure the storage and maintenance components of the WM by deemphasizing the manipulation of the material. However, Digits backward (DB) requires a transformation to reorder the input verbal digits into a reversed sequence and is believed to involve the phonological loop (phonological store and rehearsal process) and the central executive system in the putative WM model. DB is thought to be a sensitive measure of WM and has been widely used in neuropsychological research and clinical evaluations [17]–[19]. However, the neural correlates of DB capacity have not been well established.

Several studies have been performed to investigate the relationship between the gray matter volume (GMV) and the performance of the digit-span test in different populations using voxel-based morphometry (VBM) analysis. In 109 healthy elderly people, the performance of the digit-span test was positively correlated with the gray matter ratio, i.e., the GMV divided by the intracranial volume [20]. In a study of 34 normal and 40 dyslexic adults, researchers identified a region in the left posterior superior temporal sulcus where the gray matter density was positively correlated with the performance of DF and DB [16]. A study of 58 patients with neurodegenerative diseases found that the DB scores were correlated with the GMVs of the DLPFC and the inferior parietal lobule [21]. However, there is a lack of studies with large sample sizes that investigate the structural correlates of DB capacity in healthy young adults. Furthermore, none of the previous studies have substantiated the question using rsFC analysis, which investigates the correlations of the time series between a region of interest (ROI) and other voxels of the brain.

In the present study, using a VBM analysis across the whole brain, we firstly identified positive correlations between the GMV and the DB score in the right anterior superior temporal gyrus (STG), the right posterior STG, the left IFG and the left Rolandic operculum. Many previous studies on brain disorders have revealed that brain areas with changes in the GMV are commonly accompanied by altered rsFCs between these regions and other related brain areas [22]–[24]. For example, the hippocampus had both a reduced GMV [25] and rsFCs in patients with Alzheimer disease [26]. These findings suggest that structural (i.e., GMV) change in a brain area may be associated with rsFC alteration in this area and that the combination of VBM and rsFC analyses can improve our understanding of the pathology of brain diseases. We further hypothesize that the combination of these two methods may improve our understanding of the neural correlates of DB capacity. Thus, we also investigated the correlations between the DB scores and the rsFCs of the four clusters found in the VBM analysis.

## Methods

### Participants

A total of 324 right-handed healthy young adults participated in this study. Twenty-five of these subjects were excluded from further analysis due to unqualified images (2 subjects) or lack of behavior assessment (23 subjects). The remaining 299 healthy young adults (151 females, 148 males; mean age 22.7 years, range 18–29 years) were finally included. All of the subjects were Chinese speakers and had no history of neurologic or psychiatric disorders. The participants were recruited from universities or society and were paid for their participation. All of the participants gave written informed consent that was approved by the Medical Research Ethics Committee of Tianjin Medical University.

### Behavior assessments

The working-memory capacity was measured by the DB subtest of the digit span test in the Chinese Revised Wechsler Adult Intelligence Scale (WAIS-RC) [27]. The subjects performed the DB task outside of the scanner. In the DB test, a variable number of random digits were read aloud by the examiner at a rate of one digit per second. Then, the subject was asked to repeat the digits in the reverse order immediately. The DB test comprised nine levels of difficulty, with the digit sequences ranging from two to ten digits. Each level consisted of two trials with digit sequences of the same length. The difficulty is progressively increased by increasing the number of random digits and the test is terminated if both trials of a test item are repeated incorrectly. The DB score (2 to 10 points) was the maximum number of digits that the subject successfully repeated in the reverse order.

The handedness of the participants was evaluated by a Chinese questionnaire modified according to the Edinburgh Handedness Inventory [28]. The handedness preference for each of 10 items, namely writing, holding chopsticks, throwing, using a toothbrush, using scissors, striking a match, threading a needle, holding a hammer, holding a racket and washing the face, was assessed for each subject. If the same hand preference was expressed for all of the 10 items, the subject was regarded as strongly right- or left-handed. If a person preferred using his right or left hand for the first 6 items but used the other hand for each of the last 4 items, this person was regarded as right- or left-handed. If a subject mixed using his or her right and left hands in the first 6 items, this person was regarded as having a mixed handedness. In this situation, the writing hand determined its tendency. Taken together, the handedness was quantitatively strong right, right, mixed right, mixed left, left, strong left strong right, right, mixed right, mixed left, left, strong left scored as 1–6 for the strong right-, right-, mixed right-, mixed left-, left- and strong left-handedness, respectively.

### MRI acquisition

All of the MR images were acquired on a Signa HDx 3.0 Tesla MR scanner(GE Medical Systems). Tight but comfortable foam padding was used to minimize head motion, and ear plugs were used to reduce scanner noise. A high-resolution T1-weighted brain volume (BRAVO) 3D MRI sequence with 176 contiguous sagittal slices was performed with the scan parameters of repetition time (TR) = 8.1 ms; echo time (TE) = 3.1 ms; inversion time = 450 ms; field of view (FOV) = 256×256 mm^2^; slice thickness = 1.0 mm; no gap; flip angle (FA) = 13°; matrix = 256×256; and voxel size = 1.0 mm×1.0 mm×1.0 mm. The resting-state fMRI scans were performed by an echo planar imaging (EPI) sequence with the scan parameters of TR = 2000 ms, TE = 30 ms, FA = 90°, matrix = 64×64, FOV = 240×240 mm^2^, slice thickness = 4 mm, no gap, and voxel size = 3.8 mm×3.8 mm×4.0 mm. Each brain volume comprised 40 axial slices, and each functional run contained 180 volumes. During the fMRI scans, all of the subjects were instructed to keep their eyes closed, to relax and to move as little as possible.

### VBM analysis

The VBM analysis was performed using the Statistical Parametric Mapping (SPM8) software (http://www.fil.ion.ucl.ac.uk/spm/software/spm8). The structural MR images were segmented into gray matter (GM), white matter and cerebrospinal fluid using the standard unified segmentation model in SPM8. Next, the GM population template was generated from the entire image dataset using the Diffeomorphic Anatomical Registration Through Exponentiated Lie algebra (DARTEL) technique [29]. After an initial affine registration of the GM DARTEL template to the tissue probability map in Montreal Neurological Institute (MNI) space (http://www.mni.mcgill.ca/), non-linear warping of the GM images to the DARTEL GM template was performed in MNI space with 1.5-mm cubic resolution (as recommended for the DARTEL procedure). The GMV of each voxel was obtained through modulation by multiplying the GM concentration map by the non-linear determinants derived from the spatial normalization step. Finally, to compensate for the residual anatomical differences, the GMV images were smoothed with an FWHM kernel of 8 mm^3^. In effect, the analysis of the modulated data tests for regional differences in the absolute volume of the brain and eliminates the confounding effects of different individual brain sizes. After spatial pre-processing, the smoothed, modulated and normalized GMV maps were used for the statistical analysis.

A voxel-based partial correlation analysis was conducted to test the relationships between the GMVs and the DB scores, and age, gender and years of education were entered as covariates of no interest. The purpose of partial correlation is to find the unique variance between two variables while eliminating the variance from the controlling variables. Converging evidence from a number of studies across several cognitive task domains, including WM, suggests that age, gender and years of education are related to cognitive functions, such as WM [30]–[33]. Moreover, the GMV and the rsFC are correlated with age [20], [34]–[36]. Thus, we calculated partial correlation coefficients between the GMV or rsFC and the DB scores to remove the effects of age, gender and years of education.

The correction for multiple comparisons was performed using a Monte Carlo simulation. A corrected threshold of *P*<0.05 was derived from a combined threshold of *P*<0.001 for each voxel and a cluster size >147 voxels, which was determined by the AlphaSim program in the AFNI software (parameters: single voxel *P* = 0.001, 5000 simulations, FWHM = 8 mm, cluster connection radius = 2.5 mm, with gray matter mask, http://afni.nimh.nih.gov/).

### Resting-state functional connectivity analysis

The preprocessing and statistical analysis of the functional images were conducted using SPM8 (http://www.fil.ion.ucl.ac.uk/spm/software/spm8). The first 10 volumes of each functional time series were discarded for the magnetization equilibrium. The remaining 170 images were corrected for the time delay between the different slices and realigned to the first volume. The head motion parameters were computed by estimating the translation in each direction and the angular rotation on each axis for each volume, which provided a record of the head position. The realigned images were spatially normalized to the MNI EPI template and re-sampled to a 2-mm cubic voxel. The normalized images were smoothed with a Gaussian kernel of 6 mm full-width at half maximum. We required the head motions of all of the subjects to be lower than the thresholds of 2 mm of translation in each of the x, y and z directions and 2° of rotation in each of the x-, y- and z-axes; only 282 subjects satisfied this requirement and were included in the rsFC analysis. Several sources of spurious variances, including the estimated motion parameters, linear drift, global average BOLD signals, and average BOLD signals in the ventricular and white matter regions, were removed from the data through linear regression [37], [38]. Finally, temporal band-pass filtering (0.01∼0.08 Hz) was performed on the time series of each voxel to reduce the effects of low-frequency drift and high-frequency noises [37], [39].

The ROIs were defined as the brain areas in which GMVs showed significant correlations with the DB scores. Thus, four ROIs were extracted and resampled into 2-mm cubic voxels. Next, ROI-based FC analysis was performed to calculate the FC maps of each ROI using the DPARSF software (http://www.restfmri.net). For each subject, the correlation coefficients between the mean time series of each ROI and that of each voxel of the whole brain were computed and converted to *z*-values, using Fisher's *r*-to-*z* transformation to improve the normality. Subsequently, individuals' *z*-values were entered into a random-effect one-sample *t*-test in a voxel-wise manner to identify the brain regions that showed significant positive correlations with the seed region. The significant FC maps were corrected for multiple comparisons using the Family Wise Error (FWE, *P*<0.05) method. Next, a mask that included the brain areas with significant positive rsFCs with the seed regions was generated for the following correlation analyses; the brain areas with negative rsFCs were excluded because their functional significance is still under debate [40], [41].

Similar to the VBM analysis, the voxel-based partial correlation analysis was performed to test the relationships between the rsFCs of each of the four ROIs and the DB scores, and age, gender, and years of education were entered as covariates of no interest. The correction for multiple comparisons was performed using a Monte Carlo simulation. A corrected threshold of *P*<0.05 was derived from a combined threshold of *P*<0.01 for each voxel and a cluster size >110 voxels which was determined by the AlphaSim program in the AFNI software (parameters: single voxel *P* = 0.01, 5000 simulations, FWHM = 6 mm, cluster connection radius = 3.3 mm, with gray matter mask, http://afni.nimh.nih.gov/).

## Results

### Behavioral data

The demographic and behavioral data are reported in Table 1. A total of 299 healthy young adults (151 females, 148 males; mean age 22.7 years, range 18–29 years) were finally included in the VBM analysis. Among these adults, 282 subjects participated in the rsFC analysis. The mean DB score was 7 with a standard deviation of 1.5 and a range from 3 to 10. There were no significant correlations between the DB scores and the age or years of education (*P*>0.05). There was no significant difference in the DB scores between the male and female subjects (*P*>0.05).

**Table 1:** The demographic and behavioral characteristics of subjects.

Items	Mean	SD	Range
**VBM analysis**			
Male/female	148/151	—	—
Age (years)	22.7	2.4	18–29
Years of education	15.5	2.2	9–23
Handedness score	1.6	0.8	1–3
Digits backward span	7.0	1.5	3–10
**rsFC analysis**			
Male/female	137/145	—	—
Age (years)	22.7	2.4	18–29
Years of education	15.6	2.1	9–23
Handedness score	1.6	0.8	1–3
Digits backward span	7.0	1.6	3–10

Abbreviations: rsFC, resting-state functional connectivity; SD, standard deviation; VBM, voxel-based morphometry.

### VBM analysis

**Figure 1:**
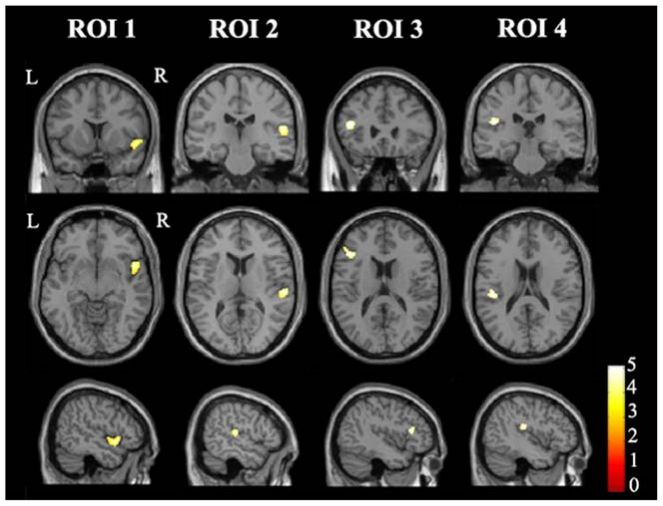
The brain areas whose GMVs are correlated with the DB scores. A total of four brain regions show positive correlations between their GMVs and the DB scores (*P*<0.05, corrected). ROIs 1–4 represent the four regions and are used as the seed regions for the rsFC analysis. Abbreviations: DB, digits backward; GMV, gray matter volume; L, left; R, right; ROI, region of interest; rsFC, resting-state functional connectivity.

The results of the correlations between the DB scores and the regional GMVs, adjusting for age, gender and years of education, are reported in Table 2 and Figure 1. No negative correlations were found between the regional GMVs and the DB scores. However, the VBM analysis revealed that the DB scores were positively correlated with the GMVs in four clusters (*P*<0.05, corrected). One cluster (ROI 1) was located in the right anterior STG (peak MNI coordinates of x = 55.5, y = 15, z = −4.5). Another cluster (ROI 2) was located in the right posterior STG (peak MNI coordinates of x = 58.5, y = −22.5, z = 13.5), a counterpart of the putative Wernicke's area in the left hemisphere. A third cluster (ROI 3) was located in the left IFG, corresponding to the putative Broca's area (peak MNI coordinates of x = −43.5, y = 25.5, z = 15). The last cluster (ROI 4) was located in the left Rolandic operculum (peak MNI coordinates of x = −45, y = −25.5, z = 19.5), which is an area that is involved in speech.

**Table 2:** Brain areas whose GMVs are correlated with the DB scores.

ROIs	Brain areas	Brodmann areas	Cluster size	Peak MNI			Peak t values
				x	y	z	
ROI 1	Right anterior STG	22, 38	465	55.5	15.0	−4.5	5.04
ROI 2	Right posterior STG	22, 42	257	58.5	−22.5	13.5	4.35
ROI 3	Left IFG	44, 45	216	−43.5	25.5	15.0	4.14
ROI 4	Left Rolandic operculum	43	179	−45.0	−25.5	19.5	4.04

Abbreviations: DB, digits backward; GMV, gray matter volume; IFG, inferior frontal gyrus; MNI, Montreal Neurological Institute; ROI, region of interest; STG, superior temporal gyrus.

### Resting-state functional connectivity analysis

**Figure 2:**
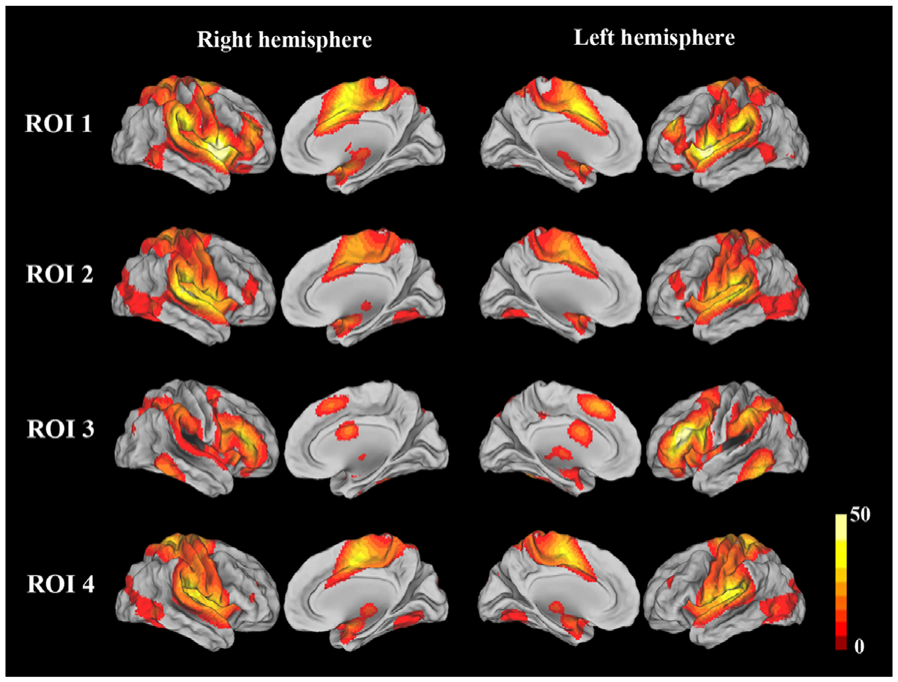
The brain areas that show positive rsFCs with each ROI (FWE corrected, *P*<0.05). The representation of each ROI is shown in Table 1. Abbreviations: FWE, family-wise error; ROI, region of interest; rsFC, resting-state functional connectivity.

**Figure 3:**
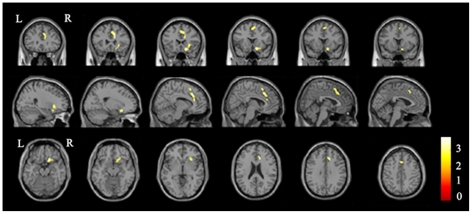
The brain areas whose rsFCs with ROI 1 are positively correlated with the DB scores (*P*<0.05, corrected). Abbreviations: DB, digits backward; L, left; R, right; ROI, region of interest; rsFC, resting-state functional connectivity.

**Figure 4:**
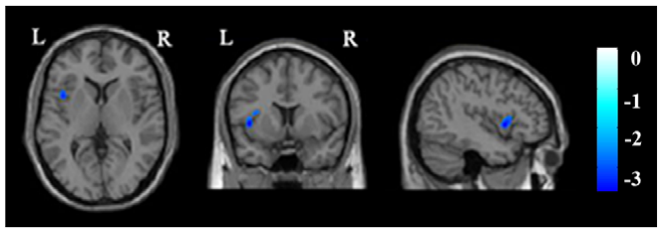
The brain areas whose rsFCs with ROI 2 are negatively correlated with the DB scores (*P*<0.05, corrected). Abbreviations: DB, digits backward; L, left; R, right; ROI, region of interest; rsFC, resting-state functional connectivity.

The four seed regions (ROIs) where the GMVs were positively correlated with the DB scores were selected for the rsFC analysis. The brain areas that had positive rsFCs with each ROI (FWE corrected, *P*<0.05) are shown in Figure 2. A partial correlation analysis was performed between the rsFCs of each ROI and the DB scores, and the results are shown in Table 3 and Figure 3. The DB scores were positively correlated with the rsFCs of ROI 1 (the right anterior STG) with the dACC (peak MNI coordinates x = 14, y = 28, z = 20) and the right fronto-insular cortex (FIC) (peak MNI coordinates x = 16, y = 20, z = −16). However, no significant negative correlations were observed for ROI 1. In contrast, the DB scores were negatively correlated with the rsFC between ROI 2 (the right posterior STG) and the left insula (peak MNI coordinates x = −38, y = 14, z = 12) (Figure 4). No significant positive correlations were observed for ROI 2. No significant correlations were observed between the DB scores and the rsFCs of ROI 3 and ROI 4.

**Table 3:** Brain areas whose rsFCs with the seed ROIs are correlated with the DB scores.

Seedregions	Connectedregions	Brodmann areas	Cluster size	Peak MNI			Peak t values
				x	y	z	
ROI 1	Right FIC	13, 47	217	16	20	−16	3.85
ROI 1	Right dACC	24,32	323	14	28	20	3.77
ROI 2	Left insula	13	111	−38	14	12	−3.59

Abbreviations: dACC, dorsal anterior cingulate cortex; DB, digits backward; FIC, fronto-insular cortex; GMV, gray matter volume; MNI, Montreal Neurological Institute; ROI, region of interest; rsFC, resting-state functional connectivity.

## Discussion

The capacity of the auditory-verbal-digital WM is dependent on the close cooperation of several subsystems, including the auditory-phonological perception and storage system, the subvocal rehearsal system, and the central executive system [2], [6], [7]. We will discuss our findings from the VBM and rsFC analyses within this framework of the verbal WM.

### The VBM analysis

#### The phonological perception and storage system

The perception of auditory input is critically important for the subsequent storage, rehearsal and executive control processes and thus relates to the verbal WM capacity as assessed by the DB test. The anterior STG belongs to the “what” pathway of the auditory system and serves for processing auditory object identity [42], [43]. The right anterior STG has been reported to respond to increased spectral variation of auditory input [44], [45], to enhance the response to auditory selective attention tasks [46], and to process auditory information about prototypicality [47]. We found that the verbal-digital WM capacity was positively correlated with the GMVs of the right anterior STG, suggesting that the accurate perception of auditory input is important for the capacity of the verbal WM. This assertion is supported by functional imaging findings indicating that better language performance is associated with more involvement of the right hemisphere [48], [49] and that higher verbal intelligence was associated with the activation of the right anterior STG [50]. A VBM study found that Chinese speakers had greater gray and white matter density in the right anterior STG compared with those who did not speak Chinese [51], which is also consistent with our finding in the Chinese population. Moreover, in patients with aphasic stroke, the language performance and outcome are found to be associated with the activation [52] and functional connectivity [53] of the right anterior STG.

The posterior STG is involved in phonological processing [54]–[57]. The left posterior STG is regarded as a “receptive” area for the analysis and identification of auditory verbal stimuli [58]. In a study of 210 stroke patients, the authors found that the structural integrity of the left posterior STG can predict the auditory short-term memory capacity [59]. It has also been shown that the gray matter density in the left posterior STG is correlated with the auditory short-term memory capacity in both normal and developmentally dyslexic groups [16]. In the present study, we found that the GMV of the right posterior STG can predict the performance of the verbal WM, which may be explained as follows. One explanation is that the right posterior STG serves certain special functions that are important for the verbal WM. This theory is supported by the findings that the right posterior STG is the main locus for the perception of sounds [60], is the preferred hemisphere for the processing of spectrotemporal auditory information [61], [47], and serves to enhance the response to auditory selective attention tasks [46]. Although the left posterior STG is the main location of activation during phonological processing, the right posterior STG is additionally recruited during phonological processing in subjects with higher performance on verbal tests. Thus, an alternative explanation is that the recruitment and structural organization of the right posterior STG reflect a capacity for language processing. This idea is consistent with the findings that the right hemispherical STG is more strongly activated in subjects with better language performance [48], [49] and higher verbal intelligence [50].

#### The subvocal rehearsal system

The left IFG (Broca's area) and the Rolandic operculum are the main components of the subvocal rehearsal system in the verbal WM model. The continuous subvocal rehearsal is critically important for the revival and maintenance of the memory trace and thus contributes to the performance of the verbal WM [2], [6]. The involvement of Broca's area in the subvocal rehearsal system of the phonological loop was first proposed by Paulesu and colleagues (1992) [10]. Subsequently, functional imaging studies on the verbal WM revealed the neural substrates of the rehearsal of phonological information in Broca's area and related motor areas [11]–[13], [62]. A functional MRI study on the DF and DB tasks revealed that the DB task additionally recruited Broca's area (BA44), in contrast to the DF task [63]. These findings are consistent with our finding that the GMV of Broca's area (BA44/45) was positively correlated with the individual verbal WM capacity, suggesting that the structural organization of the rehearsal component is one of the restricting factors of the verbal WM capacity. The left Rolandic operculum plays a role in speech production and phonological rehearsal [64], which is supported by findings that lesions in this area are associated with articulatory disorders [65] and the finding that structural and functional abnormalities of this area are associated with stuttering [66], [67].

### The rsFC analysis

#### The insular rsFC

The insula is a cytoarchitectonically and functionally heterogeneous region that has been divided into 2 or 3 subregions [68]–[70]. Resting-state fMRI has shown that each insular subregion has a distinct rsFC pattern, is involved in different functional networks, and serves a different function [71], [72]. The ventral anterior insula is primarily connected with the pregenual ACC and is involved in emotional processing; the dorsal anterior insula is connected with the dorsal ACC and is implicated in cognitive control; and the posterior insula is connected with the primary and secondary sensorimotor cortices and participates in sensorimotor processing [72]. In another parcellation scheme, the insula is divided into anterior and posterior parts, which form two mutually exclusive and anti-correlated functional networks, i.e., increases in the BOLD signal for one network correspond to decreases in the other one [71]. The anterior insula network is also called the salience network (SN). As illustrated in Figure 2, ROI 1 (the right anterior STG) is involved in the SN, whereas ROI 2 (the right posterior STG) and ROI 4 (left Rolandic operculum) belong to the posterior insula network.

#### The salience network

The SN is mainly comprised of the FIC and dorsal ACC and serves to identify the subjective salience across several domains, whether it is cognitive, homeostatic, or emotional [67], [73]–[80]. Specifically, the FIC serves to mark salient stimuli transiently from the vast and continuous stream of visual, auditory, tactile and other sensory inputs and to initiate attentional control signals, which are then sustained by the dorsal ACC and the ventrolateral and dorsolateral prefrontal cortices [74]. Once such a stimulus is detected, the right FIC initiates appropriate transient control signals to engage the brain areas of the central-executive network (CEN) mediating attention, WM and other higher-order cognitive processes while disengaging the default-mode network (DMN) via the von Economo neurons (VENs) [81] with large axons that facilitate the rapid relay of FIC and dorsal ACC signals to other cortical regions [82]. Consistent with this view, event-related fMRI shows by latency analysis that the onset of signal activity in the right FIC occurs earlier compared with the activation in the CEN nodes and the deactivation in the DMN nodes [83]. More importantly, the right FIC plays a critical role in switching between the CEN and the DMN [74], [83]. In addition to its role in the perception of the salience of stimuli, the dorsal ACC has been reported to be involved in a variety of cognitive functions, such as attention control [84]–[86], conflict monitoring [87]–[89], error monitoring and detection [90]–[93], and response selection [94], [95]. The involvement of the dorsal ACC in the WM has been reported in functional imaging studies [96]–[98]. The dorsal ACC shows greater activation during cognitive tasks in subjects with a higher capacity of WM [97], [98].

Recently, many pieces of evidence suggest that several disorders, such as frontotemporal dementia, depression, and posttraumatic stress disorder are associated with the dysfunction of the SN [99]–[102]. In the rsFC analysis, we found that the DB scores were positively correlated with the rsFCs within the SN (between the right anterior STG and the dorsal ACC and right FIC), which suggests that better WM performance is related to increased rsFCs within the SN. We also found a negative correlation between the DB scores and the rsFC within the posterior insula network (between the right posterior STG and the left posterior insula), which indicates that better WM performance is associated with reduced rsFC within the posterior insula network, which is anti-correlated with the SN. Taken together, our results are consistent with the notion that different insula subregions are involved in distinct functional networks and serve different functions.

#### The negative rsFC

Negative rsFCs (anti-correlations) have been found between the task-positive network and the default-mode network [37], [38], [103], [104] and between the neural systems underlying different components of the verbal WM [105]. Greicius et al. (2003) suggested that intrinsic anti-correlated activity might relate to the differential task-related responses in these regions [38]. Fox et al. (2005) proposed that anti-correlations may serve a differentiating role in segregating neuronal processes that serve opposite goals or competing representations [37]. However, one should be cautious when interpreting anti-correlations derived from the resting-state fMRI studies because the debate remains unsettled regarding whether anti-correlations are artifacts of the global signal regression [40], [41] or whether they reflect dynamic, truly anti-correlated functional networks [106]. Thus, in the present study, we did not analyze the correlations between the DB score and the negative rsFCs of the four ROIs derived from the VBM analysis.

### A comparison between our findings and the brain activation during the DB task

Converging evidence from near-infrared optical tomography, PET and fMRI studies show that the DB task activates the brain regions associated with WM (the bilateral DLPFC and the inferior parietal lobule (IPL), the left Broca's area, and the cerebellum), attention (the ACC), and visuospatial processing (the occipital cortex) [63], [107]–[109]. The left Broca's area and the right dorsal ACC were correlated with the DB capacity in the present study and were also activated in DB tasks in previous studies [63]. However, other related brain areas were either correlated with DB capacity or activated during DB tasks, but not both. These findings supported the concept that functional imaging during the DB task measures active processing but that off-line DB capacity is a constraint on this processing and not a process itself [16]. That is, the brain areas that show DB task-induced activation and those that correlate with DB capacity are related but are not necessarily the same areas.

### Conclusions

In the present study, we found that the capacity of the auditory-verbal WM was associated with the structural organization of the brain areas related to the phonological perception and storage and the subvocal rehearsal system. We also found that this capacity is positively correlated with the rsFC between the right anterior STG (an area related to the phonological perception) and the dorsal ACC and the right FIC (two core nodes of the SN) and anti-correlated with the resting-state functional connectivity between the right posterior STG with left posterior insula (two areas included in posterior insula pattern network). These findings suggest that digital backward performance is related to the structural and functional organizations of the brain areas involving in the auditory phonological loop, the SN and its anticorrelated networks.

## References

[1] Baddeley A (1992). Working memory.. Science.

[2] Baddeley A (2003). Working memory: looking back and looking forward.. Nat Rev Neurosci.

[3] Cowan N (2008). What are the differences between long-term, short-term, and working memory?. Prog Brain Res.

[4] Baddeley AD (1986). Working memory.

[5] Baddeley AD, Hitch G, Bower GH (1974). Working memory.. The psychology of learning and motivation.

[6] Baddeley A (1996). The fractionation of working memory.. Proc Natl Acad Sci U S A.

[7] Baddeley A (2003). Working memory and language: an overview.. J Commun Disord.

[8] Becker JT, MacAndrew DK, Fiez JA (1999). A comment on the functional localization of the phonological storage subsystem of working memory.. Brain Cogn.

[9] Buchsbaum BR, D'Esposito M (2008). The search for the phonological store: from loop to convolution.. J Cogn Neurosci.

[10] Paulesu E, Frith CD, Frackowiak RS (1993). The neural correlates of the verbal component of working memory.. Nature.

[11] Smith EE, Jonides J, Marshuetz C, Koeppe RA (1998). Components of verbal working memory: evidence from neuroimaging.. Proc Natl Acad Sci U S A.

[12] Chein JM, Fiez JA (2001). Dissociation of verbal working memory system components using a delayed serial recall task.. Cereb Cortex.

[13] Smith EE, Jonides J (1998). Neuroimaging analyses of human working memory.. Proc Natl Acad Sci U S A.

[14] Salmon E, Van der Linden M, Collette F, Delfiore G, Maquet P (1996). Regional brain activity during working memory tasks.. Brain.

[15] Collette F, Van der Linden M (2002). Brain imaging of the central executive component of working memory.. Neurosci Biobehav Rev.

[16] Richardson FM, Ramsden S, Ellis C, Burnett S, Megnin O (2011). Auditory STM capacity correlates with gray matter density in the left posterior STS in cognitively normal and dyslexic.. J Cogn Neurosci.

[17] Wilde NJ, Strauss E, Tulsky DS (2004). Memory span on the Wechsler scales.. J Clin Exp Neuropsychol.

[18] Wynn RM, Coolidge FL (2009). Does greater phonological storage capacity correlate with levels of intentionality and theory of mind?. Psychol Rep.

[19] Waters GS, Caplan D (2003). The reliability and stability of verbal working memory measures.. Behav Res Methods Instrum Comput.

[20] Taki Y, Kinomura S, Sato K, Goto R, Wu K (2011). Correlation between gray/white matter volume and cognition in healthy elderly people.. Brain Cogn.

[21] Amici S, Brambati SM, Wilkins DP, Ogar J, Dronkers NL (2007). Anatomical correlates of sentence comprehension and verbal working memory in neurodegenerative disease.. J Neurosci.

[22] Gili T, Cercignani M, Serra L, Perri R, Giove F (2011). Regional brain atrophy and functional disconnection across Alzheimer's disease evolution.. J Neurol Neurosurg Psychiatry.

[23] Liao W, Xu Q, Mantini D, Ding J, Machado-de-Sousa JP (2011). Altered gray matter morphometry and resting-state functional and structural connectivity in social anxiety disorder.. Brain Res.

[24] Lui S, Deng W, Huang X, Jiang L, Ma X (2009). Association of cerebral deficits with clinical symptoms in antipsychotic-naive first-episode schizophrenia: an optimized voxel-based morphometry and resting state functional connectivity study.. Am J Psychiatry.

[25] Kesslak JP, Nalcioglu O, Cotman CW (1991). Quantification of magnetic resonance scans for hippocampal and parahippocampal atrophy in Alzheimer's disease.. Neurology.

[26] Allen G, Barnard H, McColl R, Hester AL, Fields JA (2007). Reduced hippocampal functional connectivity in Alzheimer disease.. Arch Neurol.

[27] Gong YX (1982).

[28] Oldfield RC (1971). the assessment and analysis of handness: the Edinburgh inventory.. Neuropsychologia.

[29] Ashburner J (2007). A fast diffeomorphic image registration algorithm.. Neuroimage.

[30] Vaz IA, Cordeiro PM, Macedo EC, Lukasova K (2010). Working memory in children assessed by the Brown-Peterson Task.. Pro Fono.

[31] Otero Dadín C, Rodríguez Salgado D, Andrade Fernández E (2009). Natural sex hormone cycles and gender differences in memory.. Actas Esp Psiquiatr.

[32] Wild-Wall N, Falkenstein M, Gajewski PD (2011). Age-related differences in working memory performance in a 2-back task.. Front Psychol.

[33] Diamond A, Lee K (2011). Interventions shown to aid executive function development in children 4 to 12 years old.. Science.

[34] Fair DA, Cohen AL, Dosenbach NU, Church JA, Miezin FM (2008). The maturing architecture of the brain's default network.. Proc Natl Acad Sci U S A.

[35] Damoiseaux JS, Rombouts SA, Barkhof F, Scheltens P, Stam CJ (2006). Consistent resting-state networks across healthy subjects.. Proc Natl Acad Sci U S A.

[36] Stevens MC, Kiehl KA, Pearlson GD, Calhoun VD (2007). Functional neural networks underlying response inhibition in adolescents and adults.. Behav Brain Res.

[37] Fox MD, Snyder AZ, Vincent JL, Corbetta M, Van Essen DC (2005). The human brain is intrinsically organized into dynamic, anticorrelated functional networks.. Proc Natl Acad Sci U S A.

[38] Greicius MD, Krasnow B, Reiss AL, Menon V (2003). Functional connectivity in the resting brain: A network analysis of the default mode hypothesis.. Proc Natl Acad Sci U S A.

[39] Lowe MJ, Mock BJ, Sorenson JA (1998). Functional connectivity in single and multislice echoplanar imaging using resting-state fluctuations.. Neuroimage.

[40] Murphy K, Birn RM, Handwerker DA, Jones TB, Bandettini PA (2009). The impact of global signal regression on resting state correlations: are anti-correlated networks introduced?. Neuroimage.

[41] Weissenbacher A, Kasess C, Gerstl F, Lanzenberger R, Moser E (2009). Correlations and anticorrelations in resting-state functional connectivity MRI: a quantitative comparison of preprocessing strategies.. Neuroimage.

[42] Ahveninen J, Jääskeläinen IP, Raij T, Bonmassar G, Devore S (2006). Task-modulated “what” and “where” pathways in human auditory cortex.. Proc Natl Acad Sci U S A.

[43] Alain C, McDonald KL, Kovacevic N, McIntosh AR (2009). Spatiotemporal analysis of auditory “what” and “where” working memory.. Cereb Cortex.

[44] Zatorre RJ, Belin P (2001). Spectral and temporal processing in human auditory cortex.. Cereb Cortex.

[45] Jamison HL, Watkins KE, Bishop DV, Matthews PM (2006). Hemispheric specialization for processing auditory nonspeech stimuli.. Cereb Cortex.

[46] Petit L, Simon G, Joliot M, Andersson F, Bertin T (2007). Right hemisphere dominance for auditory attention and its modulation by eye position: an event related fMRI study.. Restor Neurol Neurosci.

[47] Lattner S, Meyer ME, Friederici AD (2005). Voice perception: Sex, pitch, and the right hemisphere.. Hum Brain Mapp.

[48] van Ettinger-Veenstra HM, Ragnehed M, Hällgren M, Karlsson T, Landtblom AM (2010). Right-hemispheric brain activation correlates to language performance.. Neuroimage.

[49] Yeatman JD, Ben-Shachar M, Glover GH, Feldman HM (2010). Individual differences in auditory sentence comprehension in children: An exploratory event-related functional magnetic resonance imaging investigation.. Brain Lang.

[50] Lidzba K, Schwilling E, Grodd W, Krägeloh-Mann I, Wilke M (2011). Language comprehension vs. language production: Age effects on fMRI activation.. Brain Lang.

[51] Crinion JT, Green DW, Chung R, Ali N, Grogan A (2009). Neuroanatomical markers of speaking Chinese.. Hum Brain Mapp.

[52] Crinion J, Price CJ (2005). Right anterior superior temporal activation predicts auditory sentence comprehension following aphasic stroke.. Brain.

[53] Warren JE, Crinion JT, Lambon Ralph MA, Wise RJ (2009). Anterior temporal lobe connectivity correlates with functional outcome after aphasic stroke.. Brain.

[54] Turkeltaub PE, Coslett HB (2010). Localization of sublexical speech perception components.. Brain Lang.

[55] Peeva MG, Guenther FH, Tourville JA, Nieto-Castanon A, Anton JL (2010). Distinct representations of phonemes, syllables, and supra-syllabic sequences in the speech production network.. Neuroimage.

[56] Graves WW, Grabowski TJ, Mehta S, Gupta P (2008). The left posterior superior temporal gyrus participates specifically in accessing lexical phonology.. J Cogn Neurosci.

[57] Burton MW, Locasto PC, Krebs-Noble D, Gullapalli RP (2005). A systematic investigation of the functional neuroanatomy of auditory and visual phonological processing.. Neuroimage.

[58] Heim S, Friederici AD (2003). Phonological processing in language production: time course of brain activity.. Neuroreport.

[59] Leff AP, Schofield TM, Crinion JT, Seghier ML, Grogan A (2009). The left superior temporal gyrus is a shared substrate for auditory short-term memory and peech comprehension: evidence from 210 patients with stroke.. Brain.

[60] Specht K, Reul J (2003). Functional segregation of the temporal lobes into highly differentiated subsystems for auditory perception: an auditory rapid event-related fMRI-task.. Neuroimage.

[61] Boemio A, Fromm S, Braun A, Poeppel D (2005). Hierarchical and asymmetric temporal sensitivity in human auditory cortices.. Nat Neurosci.

[62] Rothmayr C, Baumann O, Endestad T, Rutschmann RM, Magnussen S (2007). Dissociation of neural correlates of verbal and non-verbal visual working memory with different delays.. Behav Brain Funct.

[63] Gerton BK, Brown TT, Meyer-Lindenberg A, Kohn P, Holt JL (2004). Shared and distinct neurophysiological components of the digits forward and backward tasks as revealed by functional neuroimaging.. Neuropsychologia.

[64] Veroude K, Norris DG, Shumskaya E, Gullberg M, Indefrey P (2010). Functional connectivity between brain regions involved in learning words of a new language.. Brain Lang.

[65] Tonkonogy J, Goodglass H (1981). Language function, foot of the third frontal gyrus, and rolandic operculum.. Arch Neurol.

[66] Chang SE, Erickson KI, Ambrose NG, Hasegawa-Johnson MA, Ludlow CL (2008). Brain anatomy differences in childhood stuttering.. NeuroImage.

[67] Watkins KE, Smith SM, Davis S, Howell P (2008). Structural and functional abnormalities of the motor system in developmental stuttering.. Brain.

[68] Augustine JR (1996). Circuitry and functional aspects of the insular lobe in primates including humans.. Brain Res Rev.

[69] Craig AD (2002). How do you feel? Interoception: the sense of the physiological condition of the body.. Nat Rev Neurosci.

[70] Craig AD (2009). How do you feel—now? The anterior insula and human awareness.. Nat Rev Neurosci.

[71] Cauda F, D'Agata F, Sacco K, Duca S, Geminiani G (2011). Functional connectivity of the insula in the resting brain.. Neuroimage.

[72] Deen B, Pitskel NB, Pelphrey KA (2011). Three systems of insular functional connectivity identified with cluster analysis.. Cereb Cortex.

[73] Seeley WW, Menon V, Schatzberg AF, Keller J, Glover GH (2007). Dissociable intrinsic connectivity networks for salience processing and executive control.. J Neurosci.

[74] Menon V, Uddin LQ (2010). Saliency, switching, attention and control: a network model of insula function.. Brain Struct Funct.

[75] Menon V, Adleman NE, White CD, Glover GH, Reiss AL (2001). Error-related brain activation during a Go/NoGo response inhibition task.. Hum Brain Mapp.

[76] Curtis CE, D'Esposito M (2003). Persistent activity in the prefrontal cortex during working memory.. Trends Cogn Sci.

[77] Kerns JG, Cohen JD, MacDonald AW, Cho RY, Stenger VA (2004). Anterior cingulate conflict monitoring and adjustments in control.. Science.

[78] Ridderinkhof KR, Ullsperger M, Crone EA, Nieuwenhuis S (2004). The role of the medial frontal cortex in cognitive control.. Science.

[79] Peyron R, Laurent B, García-Larrea L (2000). Functional imaging of brain responses to pain. A review and meta-analysis.. Neurophysiol Clin.

[80] Grinband J, Hirsch J, Ferrera VP (2006). A neural representation of categorization uncertainty in the human brain.. Neuron.

[81] Nimchinsky EA, Gilissen E, Allman JM, Perl DP, Erwin JM (1999). A neuronal morphologic type unique to humans and great apes.. Proc Natl Acad Sci U S A.

[82] Allman JM, Watson KK, Tetreault NA, Hakeem AY (2005). Intuition and autism: a possible role for Von Economo neurons.. Trends Cogn Sci.

[83] Sridharan D, Levitin DJ, Menon V (2008). A critical role for the right fronto-insular cortex in switching between central-executive and default-mode networks.. Proc Natl Acad Sci U S A.

[84] Smith EE, Jonides J (1999). Storage and executive processes in the frontal lobes.. Science.

[85] Crottaz-Herbette S, Menon V (2006). Where and When the Anterior Cingulate Cortex Modulates Attentional Response: Combined fMRI and ERP Evidence.. J Cogn Neurosci.

[86] Luo Q, Mitchell D, Jones M, Mondillo K, Vythilingam M (2007). Common regions of dorsal anterior cingulate and prefrontal-parietal cortices provide attentional control of distracters varying in emotionality and visibility.. Neuroimage.

[87] Botvinick MM, Cohen JD, Carter CS (2004). Conflict monitoring and anterior cingulate cortex: an update.. Trends Cogn Sci.

[88] Carter CS, Braver TS, Barch DM, Botvinick MM, Noll D (1998). Anterior cingulate cortex, error detection, and the online monitoring of performance.. Science.

[89] Carter CS, van Veen V (2007). Anterior cingulate cortex and conflict detection: an update of theory and data.. Cogn Affect Behav Neurosci.

[90] Gehring WJ, Knight RT (2000). Prefrontal–cingulate interactions in action monitoring.. Nat Neurosci.

[91] Gehring WJ, Fencsik DE (2001). Functions of the medial frontal cortex in the processing of conflict and errors.. J Neurosci.

[92] Pourtois G, Vocat R, N'Diaye K, Spinelli L, Seeck M (2010). Errors recruit both cognitive and emotional monitoring systems: simultaneous intracranial recordings in the dorsal anterior cingulate gyrus and amygdala combined with fMRI.. Neuropsychologia.

[93] Lorist MM, Boksem MA, Ridderinkhof KR (2005). Impaired cognitive control and reduced cingulate activity during mental fatigue.. Brain Res Cogn Brain Res.

[94] Awh E, Gehring WJ (1999). The anterior cingulate cortex lends a hand in response selection.. Nat Neurosci.

[95] Paus T (2001). Primate anterior cingulate cortex: where motor control, drive and cognition interface.. Nat Rev Neurosci.

[96] Lenartowicz A, McIntosh AR (2005). The role of anterior cingulate cortex in working memory is shaped by functional connectivity.. J Cogn Neurosci.

[97] Osaka M, Osaka N, Kondo H, Morishita M, Fukuyama H (2003). The neural basis of individual differences in working memory capacity: an fMRI study.. Neuroimage.

[98] Kondo H, Morishita M, Osaka N, Osaka M, Fukuyama H (2004). Functional roles of the cingulo-frontal network in performance on working memory.. Neuroimage.

[99] Zhou J, Greicius MD, Gennatas ED, Growdon ME, Jang JY (2010). Divergent network connectivity changes in behavioural variant frontotemporal dementia and Alzheimer's disease.. Brain.

[100] Seeley WW (2010). Anterior insula degeneration in frontotemporal dementia.. Brain Struct Funct.

[101] Daniels JK, McFarlane AC, Bluhm RL, Moores KA, Clark CR (2010). Switching between executive and default mode networks in posttraumatic stress disorder: alterations in functional connectivity.. J Psychiatry Neurosci.

[102] Veer IM, Beckmann CF, van Tol MJ, Ferrarini L, Milles J (2010). Whole brain resting-state analysis reveals decreased functional connectivity in major depression.. Front Syst Neurosci.

[103] Tian LX, Jiang TZ, Liu Y, Yu CS, Wang K (2007). The relationship within and between the extrinsic and intrinsic systems indicated by resting state correlational patterns of sensory cortices.. Neuroimage.

[104] Zhou Y, Yu C, Zheng H, Liu Y, Song M (2010). Increased neural resources recruitment in the intrinsic organization in major depression.. J Affect Disord.

[105] Grube O, Muller T, Falkai P (2007). Dynamic interactions between neural systems underlying different components of verbal working memory.. J Neural Transm.

[106] Hampson M, Driesen N, Roth JK, Gore JC, Constable RT (2010). Functional connectivity between task-positive and task-negative brain areas and its relation to working memory performance.. Magn Reson Imaging.

[107] Hoshi Y, Oda I, Wada Y, Ito Y, Yutaka Yamashita (2000). Visuospatial imagery is a fruitful strategy for the digit span backward task: a study with near-infrared optical tomography.. Brain Res Cogn Brain Res.

[108] Sun X, Zhang X, Chen X, Zhang P, Bao M (2005). Age-dependent brain activation during forward and backward digit recall revealed by fMRI.. Neuroimage.

[109] Hale TS, Bookheimer S, McGough JJ, Phillips JM, McCracken JT (2007). Atypical brain activation during simple and complex levels of processing in adult ADHD: an fMRI study.. J Atten Disord.

